# Supplementary material for: Pangenome-aware DeepVariant
Source: bioRxiv. 2025 Jun 6:2025.06.05.657102. Preprint. [Version 1] doi: 10.1101/2025.06.05.657102 (PMC12157594; doi:10.1101/2025.06.05.657102)
Supplement: Supplement 2 [file NIHPP2025.06.05.657102v1-supplement-2.pdf]

bioRxiv preprint doi: https://doi.org/10.1101/2025.06.05.657102; this version posted June 6, 2025. The copyright holder for this preprint (which was not certified by peer review) is the author/funder, who has granted bioRxiv a license to display the preprint in perpetuity. It is made available under aCC-BY 4.0 International license.

Supplementary Figures

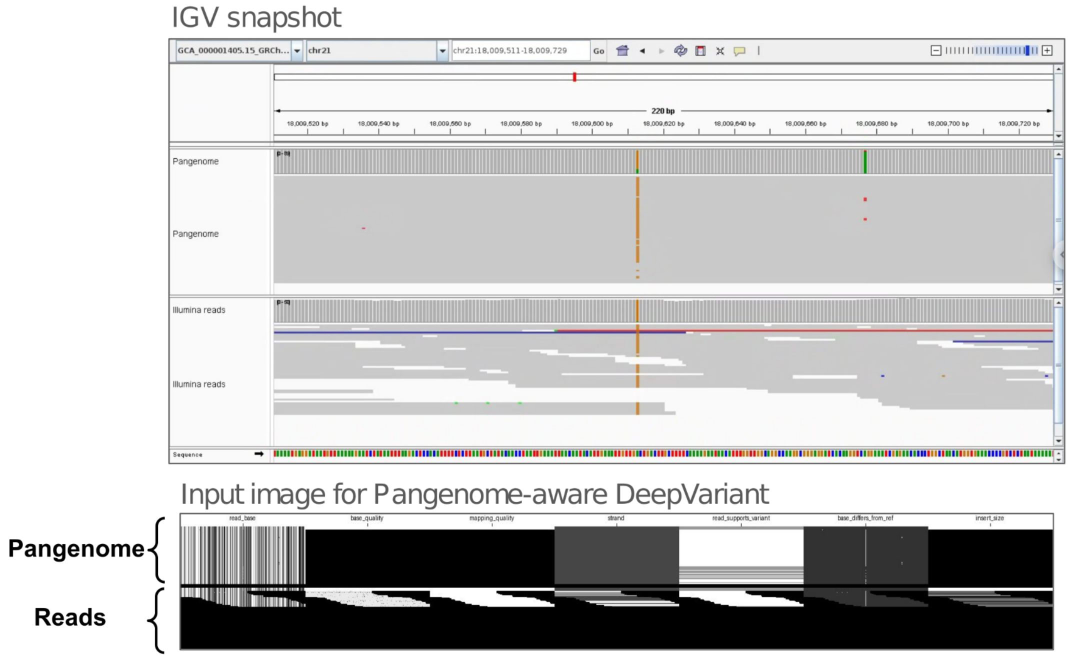

Supplementary Figure 1: Input pileup image for pangenome-aware DeepVariant.
The top panel shows an IGV snapshot with pangenome haplotypes and short read alignments. The bottom panel shows the related DeepVariant pileup image for the same window as the top IGV snapshot. Pangenome-aware DeepVariant creates this image and feeds it into the CNN model. The top and bottom parts of the pileup image contain pangenome haplotypes and read alignments respectively.

bioRxiv preprint doi: https://doi.org/10.1101/2025.06.05.657102; this version posted June 6, 2025. The copyright holder for this preprint (which was not certified by peer review) is the author/funder, who has granted bioRxiv a license to display the preprint in perpetuity. It is made available under aCC-BY 4.0 International license.

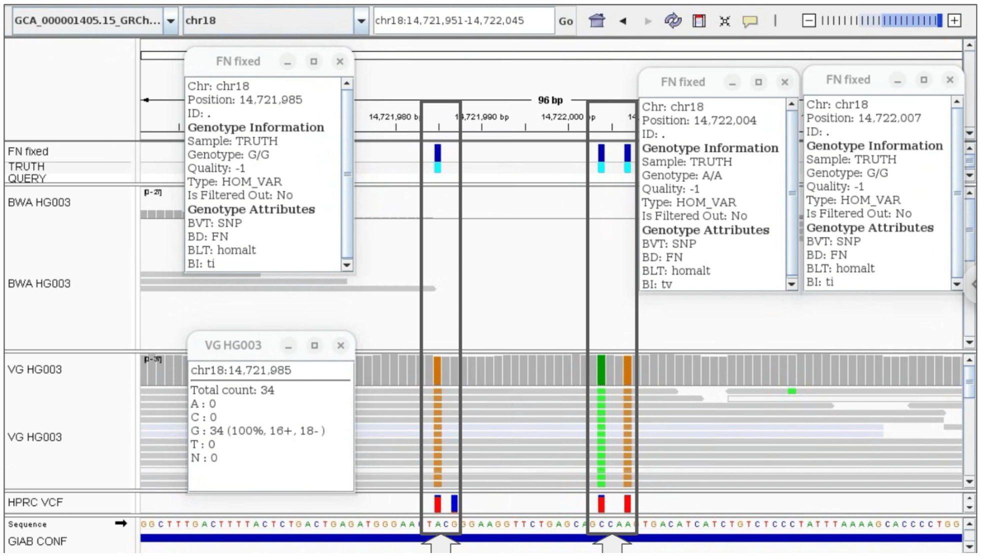

Supplementary Figure 2: Example of false negative calls rescued by using vg giraffe mappings linear-reference-based DV (with no pangenome) was run on HG003 NovaSeq reads mapped with vg giraffe and BWA-MEM. vg mappings provide clear signals for three true positive homozygous calls based on the GIAB-v4.2.1 truth set. This led to rescuing these calls which were missed when BWA-MEM was the mapper.

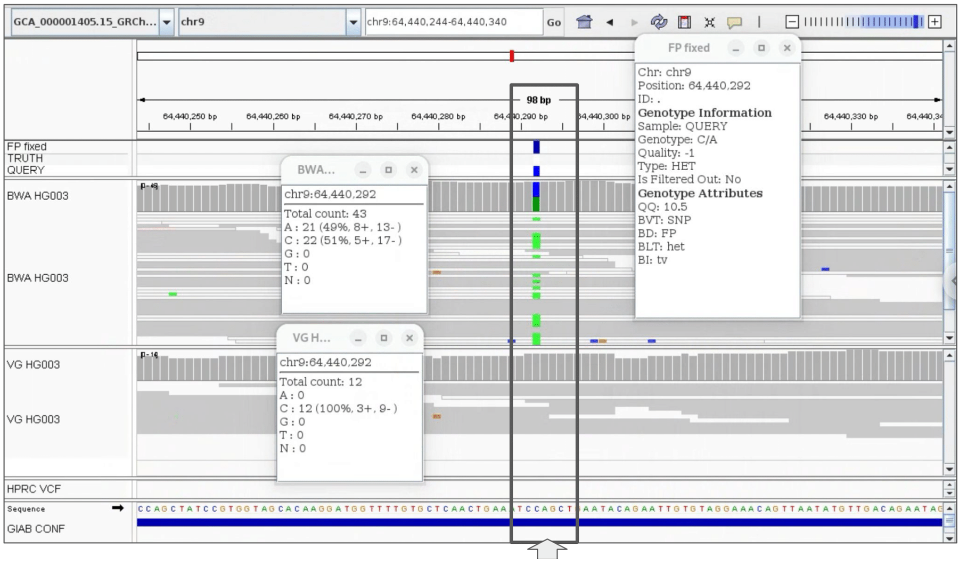

Supplementary Figure 3: Example of a false positive call removed by using vg giraffe mappings linear-reference-based DV (with no pangenome) was run on HG003 NovaSeq reads mapped with vg giraffe and BWA-MEM. Some of the BWA-MEM mappings contain a SNP however none of the vg mappings contains that. This led to calling a false positive heterozygous SNP with BWA-MEM, which was absent from the calls made with vg mappings.

bioRxiv preprint doi: https://doi.org/10.1101/2025.06.05.657102; this version posted June 6, 2025. The copyright holder for this preprint (which was not certified by peer review) is the author/funder, who has granted bioRxiv a license to display the preprint in perpetuity. It is made available under aCC-BY 4.0 International license.

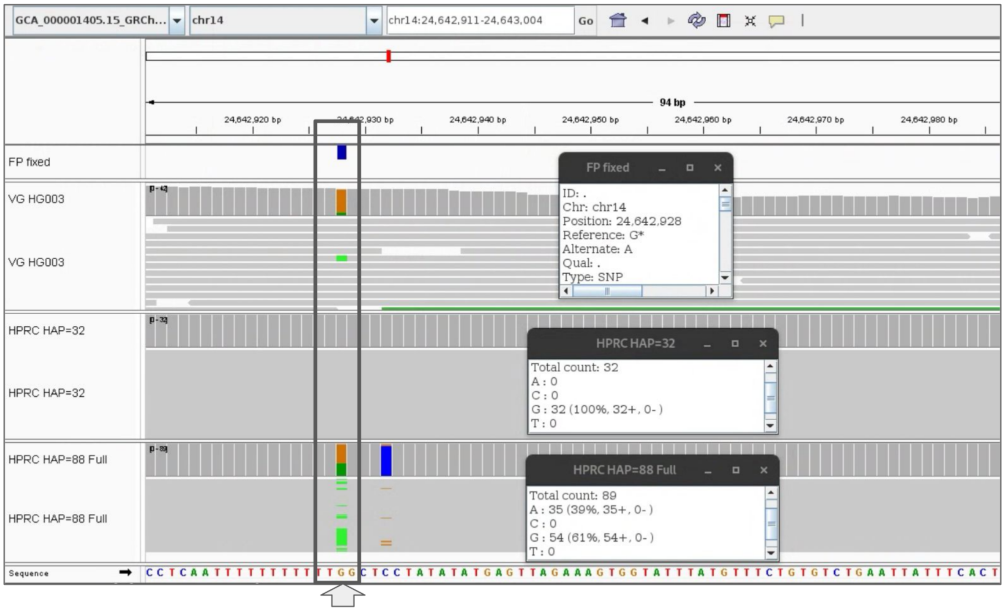

Supplementary Figure 4: Example of a false positive call removed by using personalized pangenome
Here is an example of how a personalized pangenome provides the haplotypes that more closely represent the sample's genome. In this example when pangenome-aware DV was run with full pangenome (88 haplotypes + CHM13 reference) it called a false positive; however when personalized pangenome was used (with 32 haplotypes) it didn't call it. All haplotypes with the alternative allele of "A" were removed in the personalized pangenome.

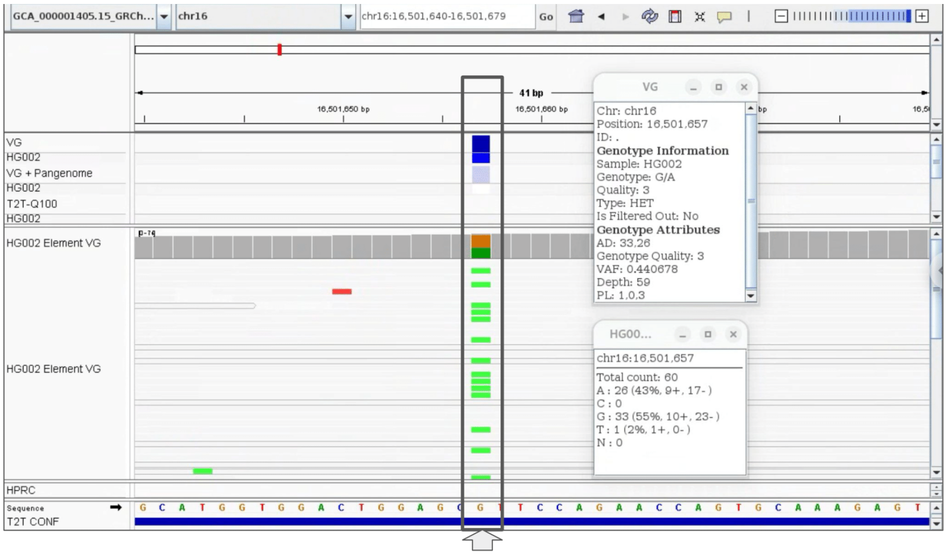

Supplementary Figure 5: Example of a false positive call removed by pangenome-aware DV
linear-reference-based DV and pangenome-aware DV were run on HG002 Element reads mapped with vg giraffe. We show one FP call in chr16 that was removed after using pangenome-aware DV. The alternative allele was completely absent from the pangenome.

bioRxiv preprint doi: https://doi.org/10.1101/2025.06.05.657102; this version posted June 6, 2025. The copyright holder for this preprint (which was not certified by peer review) is the author/funder, who has granted bioRxiv a license to display the preprint in perpetuity. It is made available under aCC-BY 4.0 International license.

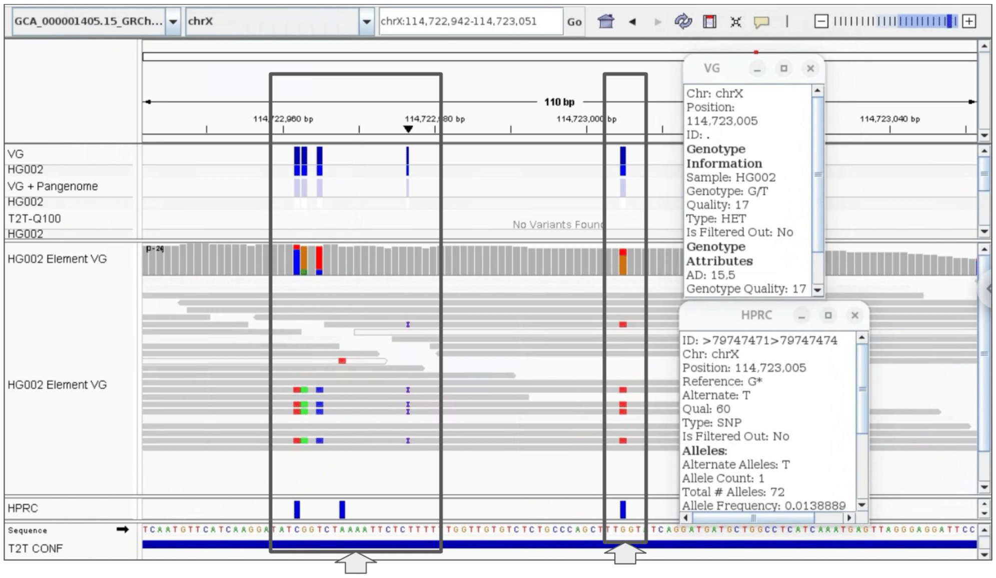

Supplementary Figure 6: Example of false positive calls removed by pangenome-aware DV linear-reference-based DV and pangenome-aware DV were run on HG002 Element reads mapped with vg. We show five FP calls in chrX that were removed after using pangenome-aware DV. For example for the right-most FP SNP there is only one haplotype (out of 72) with the alternative allele in the HPRC panel.

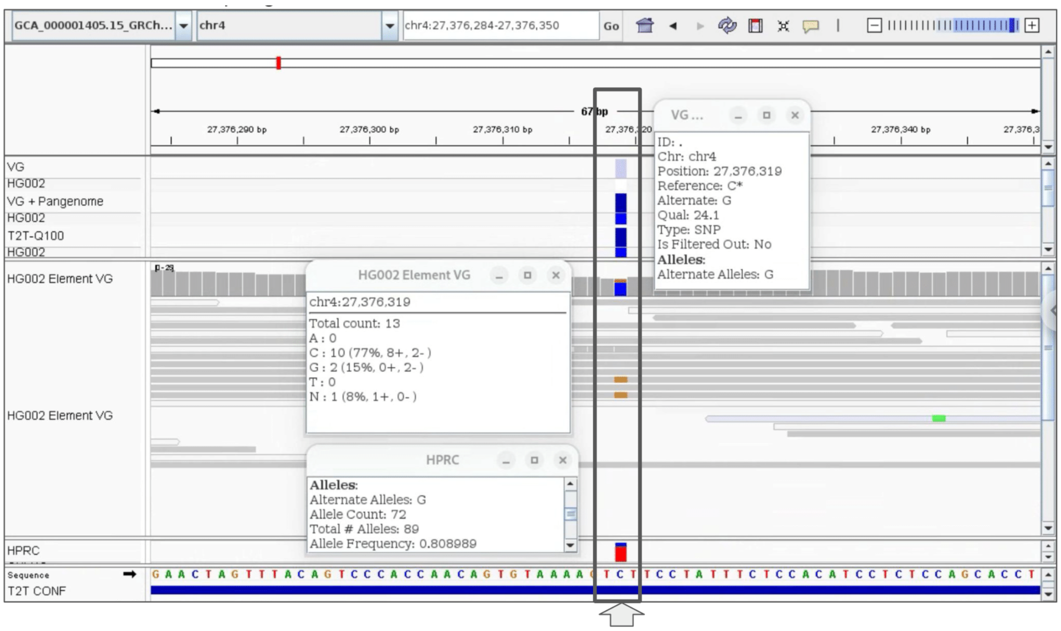

Supplementary Figure 7: Example of a false negative call rescued by pangenome-aware DV linear-reference-based DV and pangenome-aware DV were run on HG002 Element reads mapped with vg giraffe. We show one FN call in chr4 that was rescued after using pangenome-aware DV. There are 72 haplotypes (out of 89) supporting this allele in the HPRC panel.

bioRxiv preprint doi: https://doi.org/10.1101/2025.06.05.657102; this version posted June 6, 2025. The copyright holder for this preprint (which was not certified by peer review) is the author/funder, who has granted bioRxiv a license to display the preprint in perpetuity. It is made available under aCC-BY 4.0 International license.

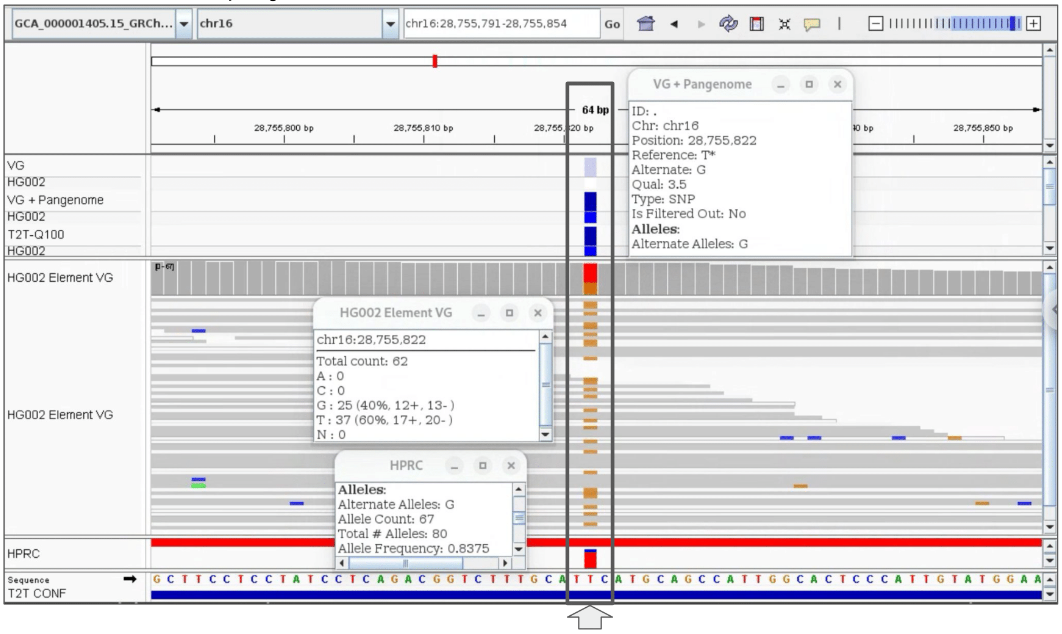

Supplementary Figure 8: Example of a false negative call rescued by pangenome-aware DV
linear-reference-based DV and pangenome-aware DV were run on HG002 Element reads mapped with vg. We show one FN call in chr16 that was rescued after using pangenome-aware DV. There are 67 haplotypes (out of 80) supporting this allele in the HPRC panel.

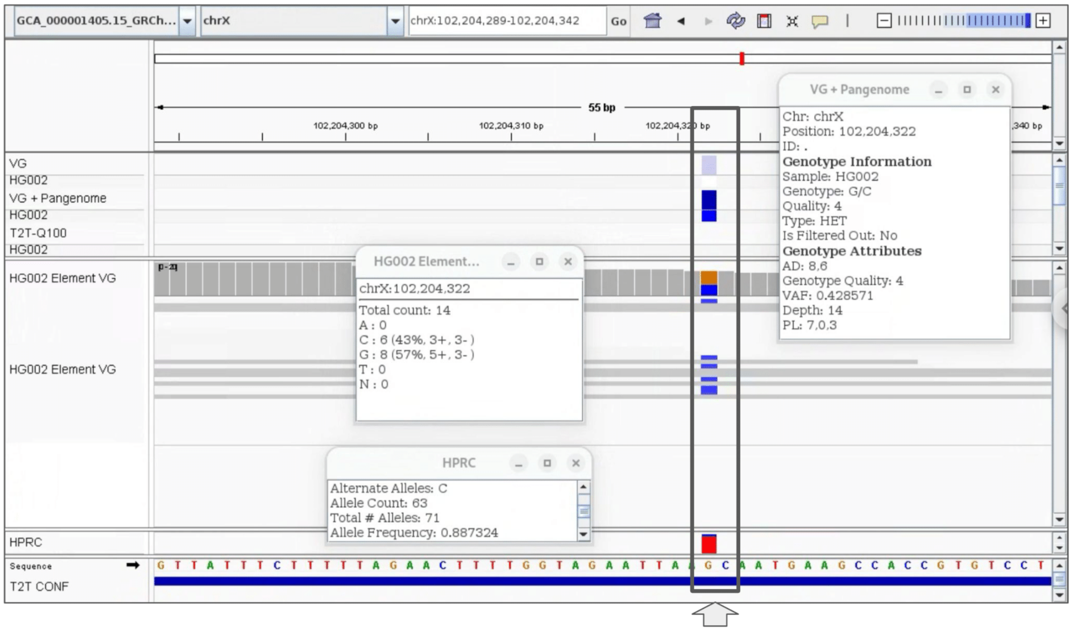

Supplementary Figure 9: Example of a false positive call induced by pangenome-aware DV
linear-reference-based DV and pangenome-aware DV were run on HG002 Element reads mapped with vg giraffe. Pangenome-aware DV calls a FP heterozygous SNP, which was not called by the linear-reference-based DV. The alternate allele “C” is highly common in the HPRC panel (63 out of 71).

bioRxiv preprint doi: https://doi.org/10.1101/2025.06.05.657102; this version posted June 6, 2025. The copyright holder for this preprint (which was not certified by peer review) is the author/funder, who has granted bioRxiv a license to display the preprint in perpetuity. It is made available under aCC-BY 4.0 International license.

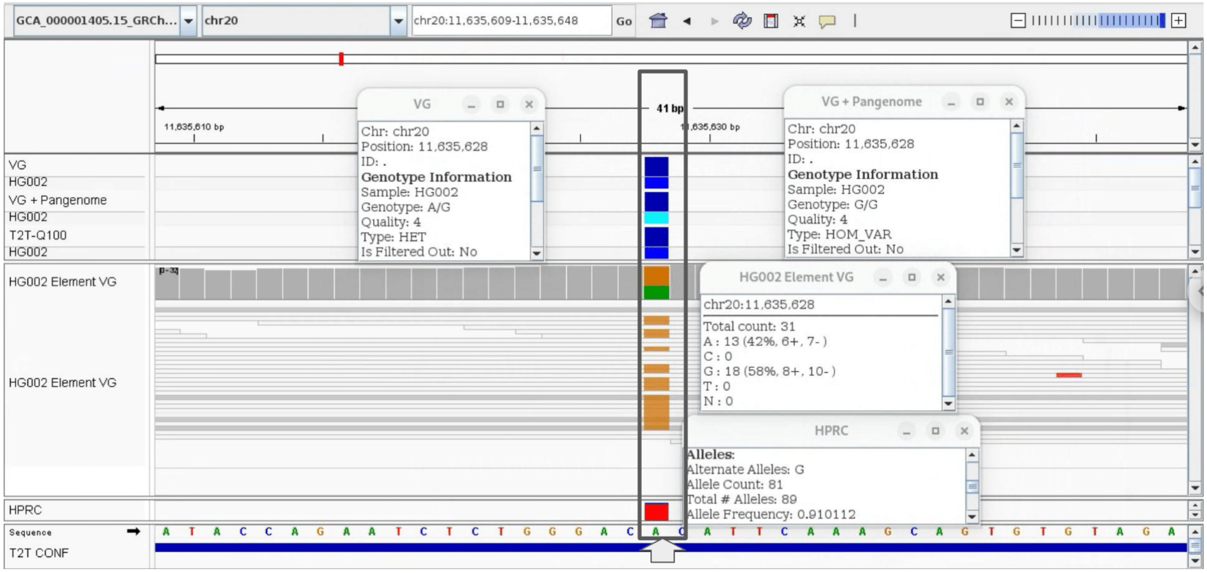

Supplementary Figure 10: Example of a false positive call induced by pangenome-aware DV linear-reference-based DV and pangenome-aware DV were run on HG002 Element reads mapped with vg giraffe. linear-reference-based DV calls a TP heterozygous SNP however pangenome-aware DV calls it homozygous by mistake. There are 81 haplotypes (out of 89) supporting this allele in the HPRC panel.

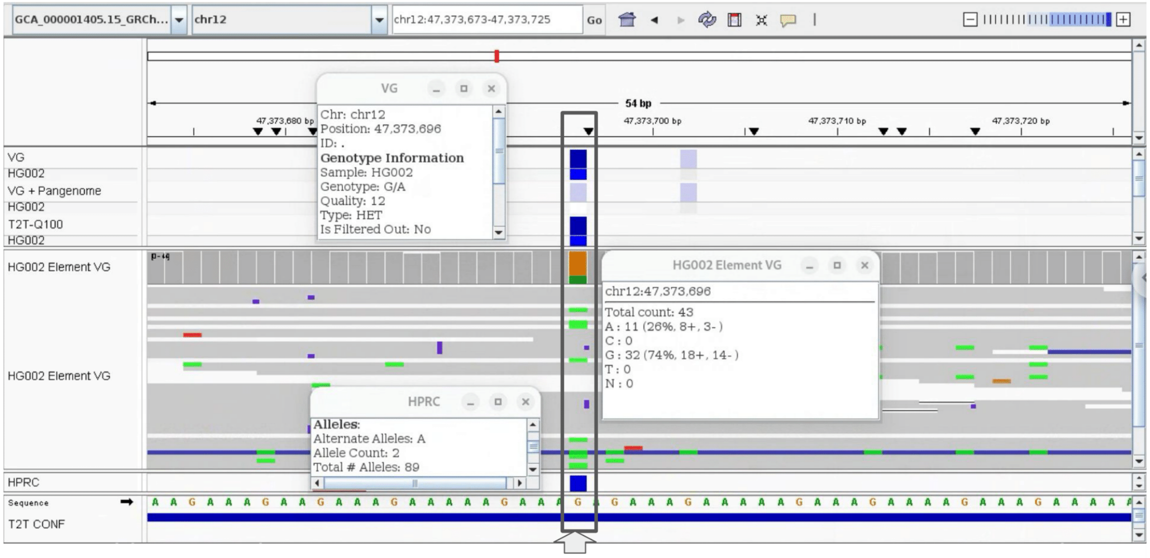

Supplementary Figure 11: Example of a false negative call induced by pangenome-aware DV linear-reference-based DV and pangenome-aware DV were run on HG002 Element reads mapped with vg giraffe. linear-reference-based DV calls a TP heterozygous SNP however it is missed by pangenome-aware DV. There are only 2 haplotypes (out of 89) supporting this allele in the HPRC panel.

bioRxiv preprint doi: https://doi.org/10.1101/2025.06.05.657102; this version posted June 6, 2025. The copyright holder for this preprint (which was not certified by peer review) is the author/funder, who has granted bioRxiv a license to display the preprint in perpetuity. It is made available under aCC-BY 4.0 International license.

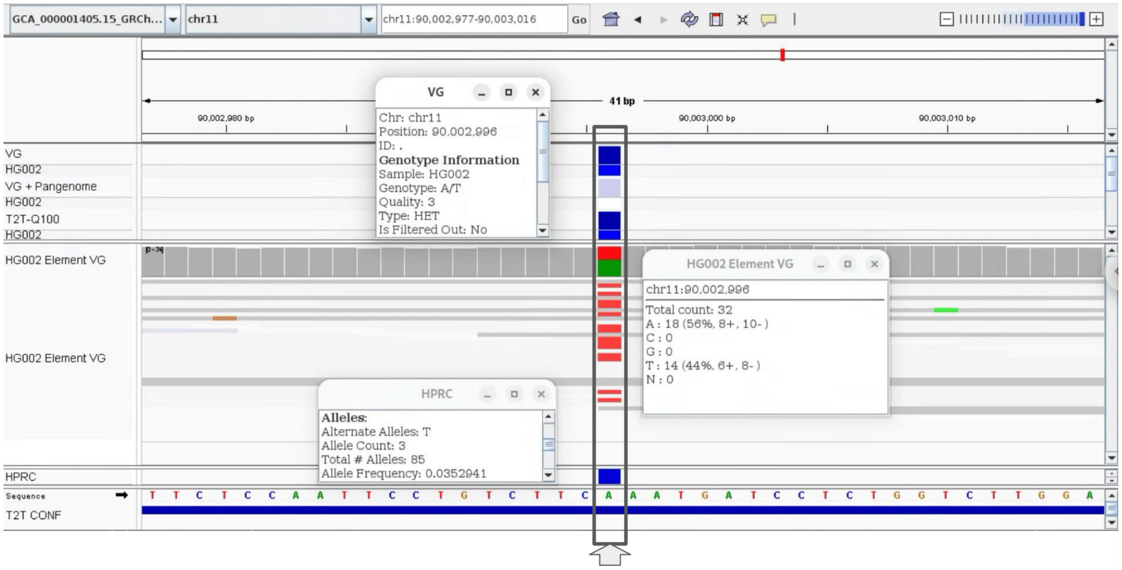

Supplementary Figure 12: Example of a false negative call induced by pangenome-aware DV
linear-reference-based DV and pangenome-aware DV were run on HG002 Element reads mapped with vg giraffe. linear-reference-based DV calls a TP heterozygous SNP however it is missed by pangenome-aware DV. There are only 3 haplotypes (out of 85) supporting this allele in the HPRC panel.

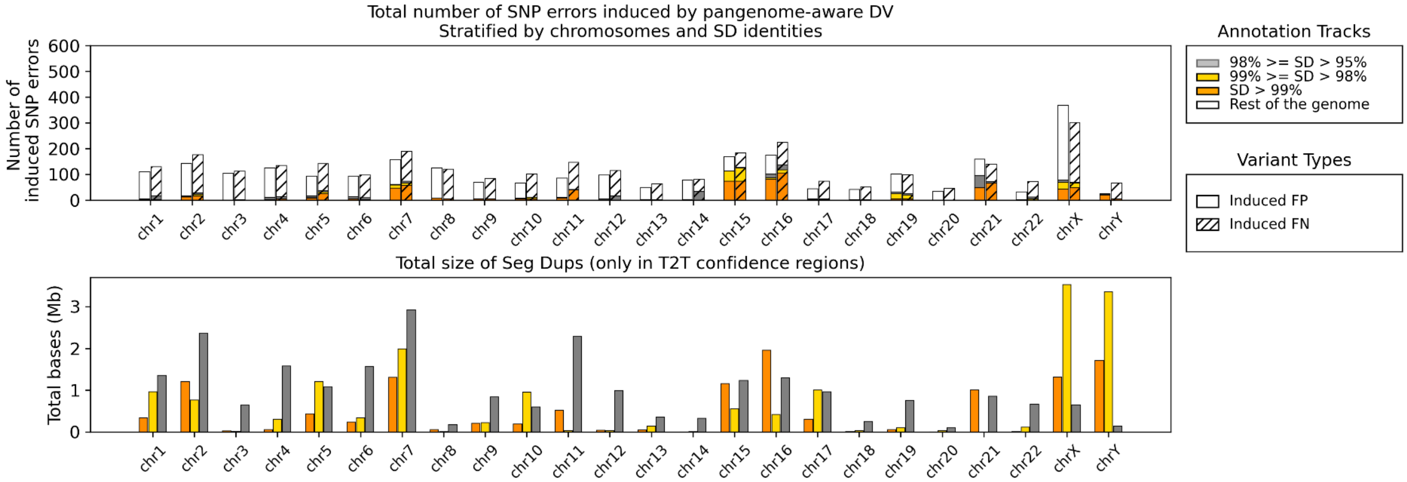

Supplementary Figure 13: Total number of SNP errors induced by pangenome-aware DV and total size of seg dups in T2T high-confidence regions
Top panel shows the number of SNP errors induced by pangenome-aware DV, which were absent from the linear-reference-based DV. For this analysis HG002 Element reads were mapped with vg giraffe and

bioRxiv preprint doi: https://doi.org/10.1101/2025.06.05.657102; this version posted June 6, 2025. The copyright holder for this preprint (which was not certified by peer review) is the author/funder, who has granted bioRxiv a license to display the preprint in perpetuity. It is made available under aCC-BY 4.0 International license.

the T2T-Q100 truth set was used for benchmarking. This panel can be compared with Figure 3d (equivalent figure but for fixed SNP errors). The bottom panel shows the total length of segmental duplications in the high-confidence bed file stratified by identity across all chromosomes.

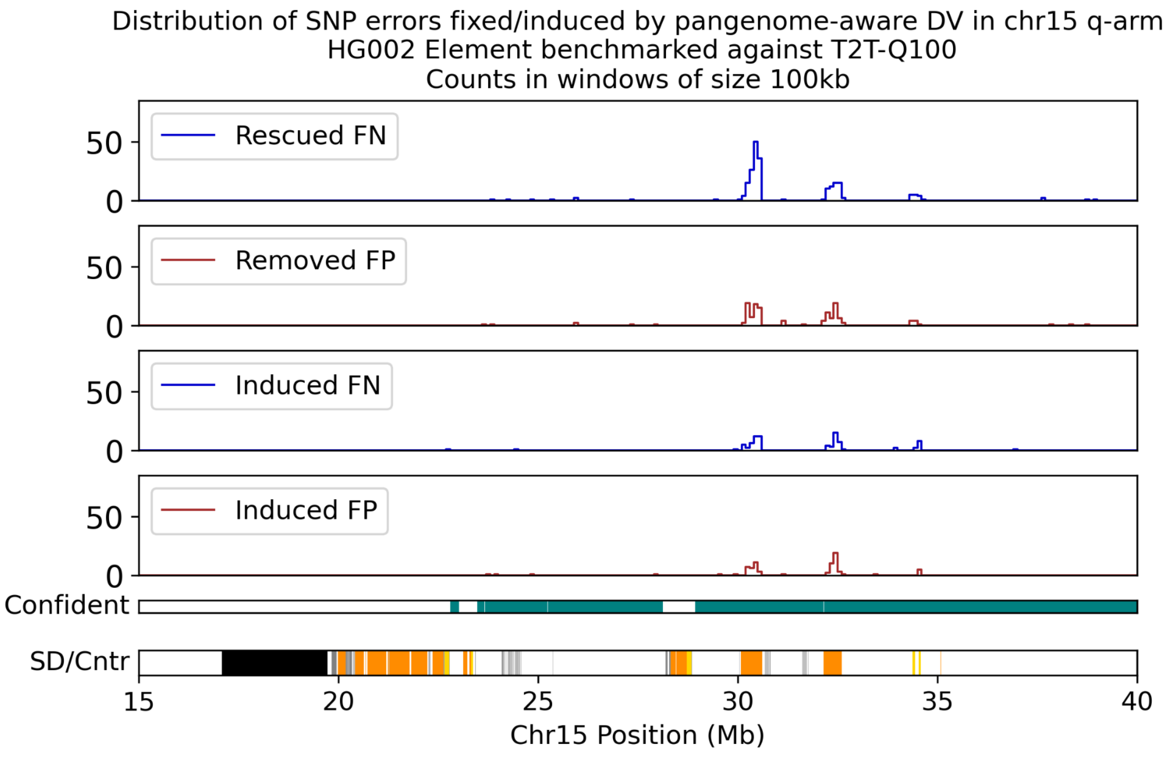

Supplementary Figure 14: Locations of SNP errors fixed or induced by pangenome-aware DV in the q-arm of chr15

This figure shows how segmental duplications are enriched with fixed variants (especially for rescued FN in this region). Density of fixed and induced errors are shown in the q-arm of chr15. The number of variants are counted in adjacent non-overlapping windows of length 100kb. Centromere is shown with black color in the bottom track.

bioRxiv preprint doi: https://doi.org/10.1101/2025.06.05.657102; this version posted June 6, 2025. The copyright holder for this preprint (which was not certified by peer review) is the author/funder, who has granted bioRxiv a license to display the preprint in perpetuity. It is made available under aCC-BY 4.0 International license.

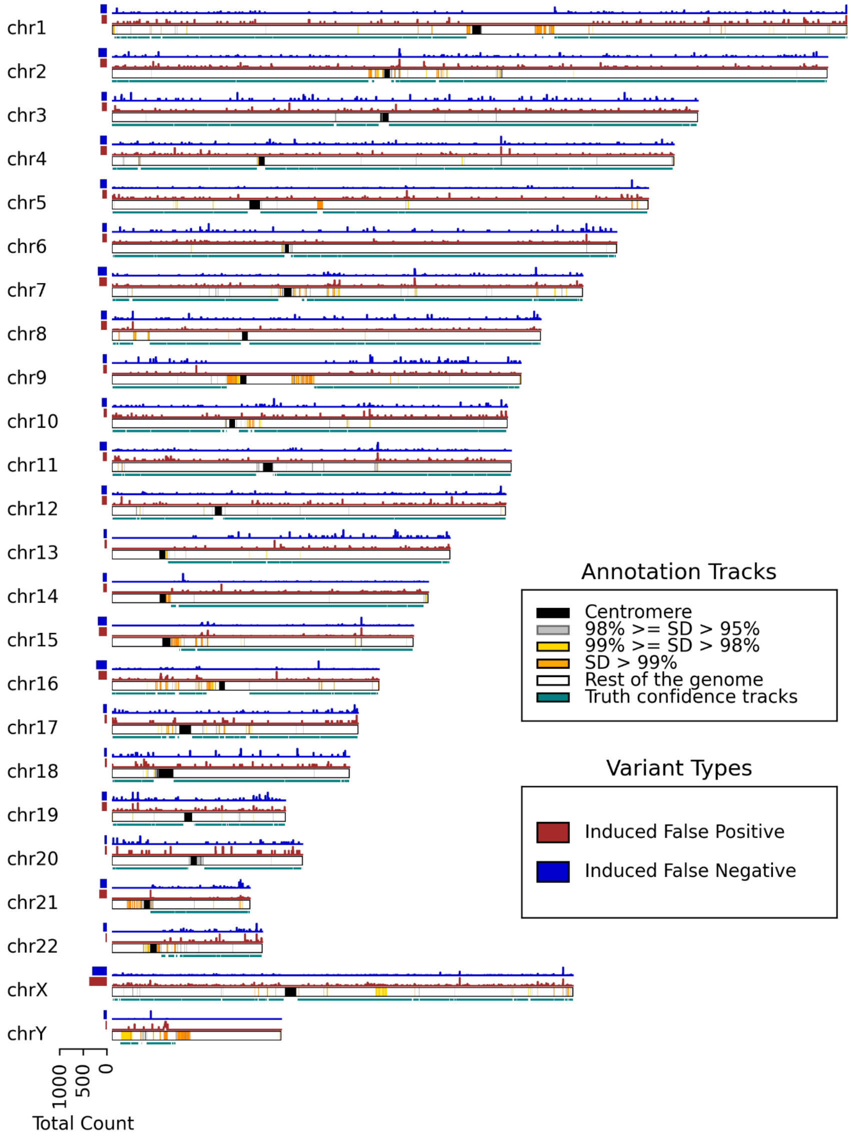

Supplementary Figure 15: Ideogram of SNP errors induced by pangenome-aware DV
The locations of the induced errors are plotted across the GRCh38 chromosomes. FP and FN calls are shown with red and blue respectively. The bottom track for each chromosome shows 5 different annotations; centromere (black), SDs with identity greater than 99% (orange), SDs with identity between 99% and 98% (yellow), SDs with identity lower than 95% (light gray) and the rest of the genome (white). Below the SD/Cntr annotation the tracks with teal color show the confidence regions for T2T-Q100 truth set. The left horizontal barplots show the total counts of induced errors per chromosome.

bioRxiv preprint doi: https://doi.org/10.1101/2025.06.05.657102; this version posted June 6, 2025. The copyright holder for this preprint (which was not certified by peer review) is the author/funder, who has granted bioRxiv a license to display the preprint in perpetuity. It is made available under aCC-BY 4.0 International license.

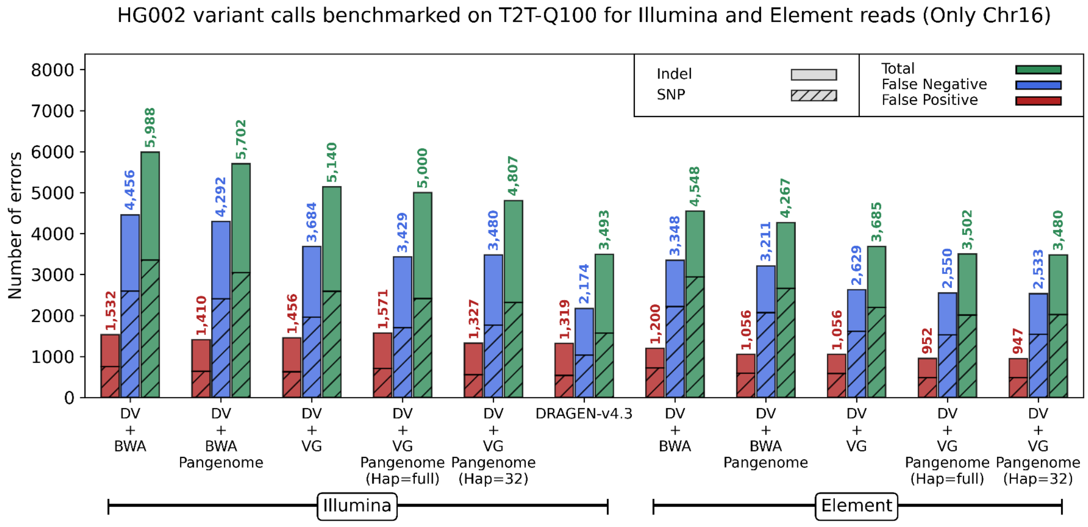

Supplementary Figure 16 : Benchmarking HG002 variant calls against T2T-Q100 on chr16
HG002 Illumina and Element calls are benchmarked against the T2T-Q100 truth set only on chromosome 16 which was the held-out chromosome for training DeepVariant. Both linear-reference-based DV and pangenome-aware DV have been tested with vg giraffe and BWA-MEM mappers. The x-axis labels with "Pangenome" refer to the pangenome-aware DV and for the rest, the linear-reference-based DV was used for variant calling. The x-axis labels with "(Hap=full)" refers to using all 88 haplotypes in the HPRC-v1.1 pangenome and "(Hap=32)" refers to using a personalized pangenome for HG002 with 32 haplotypes.

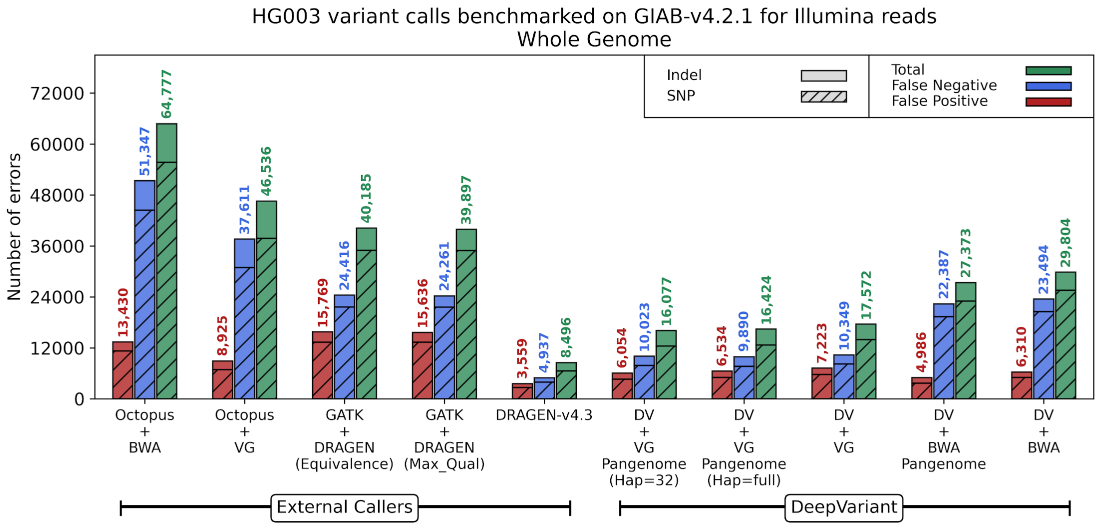

Supplementary Figure 17: Benchmarking HG003 Illumina calls against GIAB-v4.2.1 truth set.
HG003 Illumina calls are benchmarked against the GIAB-v4.2.1 truth set across the whole-genome GIAB high-confidence regions. Different modes of DeepVariant (DV) have been tested with both vg giraffe and BWA-MEM read mappers. The x-axis labels with "Pangenome" refer to the pangenome-aware DeepVariant and for the rest the linear-reference-based DeepVariant was used for variant calling. The x-axis labels with "(Hap=full)" refers to using all 88 haplotypes in the HPRC-v1.1 pangenome and "(Hap=32)" refers to using a personalized pangenome with 32 haplotypes.

bioRxiv preprint doi: https://doi.org/10.1101/2025.06.05.657102; this version posted June 6, 2025. The copyright holder for this preprint (which was not certified by peer review) is the author/funder, who has granted bioRxiv a license to display the preprint in perpetuity. It is made available under aCC-BY 4.0 International license.

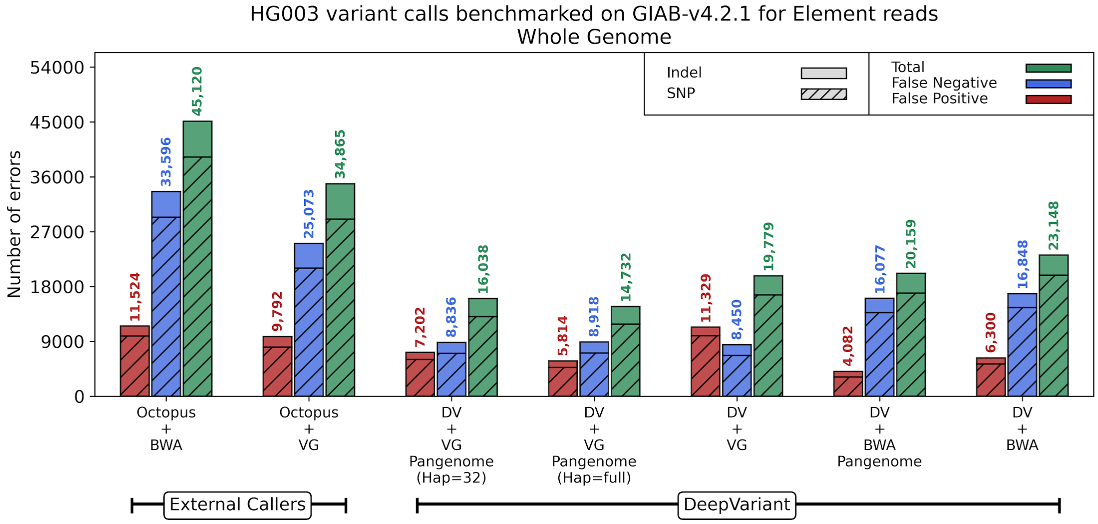

Supplementary Figure 18: Benchmarking HG003 Element calls against GIAB-v4.2.1 truth set. HG003 Element calls are benchmarked on the GIAB truth set across the whole-genome GIAB high-confidence regions. Different modes of DeepVariant (DV) have been tested with both vg giraffe and BWA-MEM read mappers. The x-axis labels with "Pangenome" refer to the pangenome-aware DeepVariant and for the rest the linear-reference-based DeepVariant was used for variant calling. The x-axis labels with "(Hap=full)" refers to using all 88 haplotypes in the HPRC-v1.1 pangenome and "(Hap=32)" refers to using a personalized pangenome with 32 haplotypes. The DRAGEN-assisted GATK failed with Element data and Dragen-v4.3 is not designed for Element so they are not reported among the external callers.
